# The impact of stroke on people living in central Uganda: A descriptive study

**DOI:** 10.4102/ajod.v7i0.438

**Published:** 2018-11-29

**Authors:** Julius T. Kamwesiga, Lena K. von Kock, Gunilla M. Eriksson, Susanne G.E. Guidetti

**Affiliations:** 1 Department of Neurobiology Care Sciences and Society, Karolinska Institute, Sweden; 2 Occupational Therapy School, Institute of Allied Health and Management Sciences - Mulago, Uganda; 3 Department of Neurology, Karolinska University Hospital, Sweden; 4 Department of Neuroscience, Rehabilitation Medicine, Uppsala University, Sweden

## Abstract

**Background:**

Knowledge about perceived impact of stroke on everyday life as well as rehabilitation needs after stroke in Uganda is necessary to identify and develop rehabilitation interventions.

**Objectives:**

To explore and describe clinical characteristics and functioning during the acute or subacute phase and chronic phase, as well as the impact of stroke on everyday life during the chronic phase in stroke survivors in central Uganda.

**Method:**

A cross-sectional observational study was conducted on a consecutively included acute or subacute (*n* = 58) sample and a chronic (*n* = 62) sample. Face-to-face interviews were conducted to collect demographic information and clinical characteristics. The Scandinavian Stroke Scale (SSS) was used to collect clinical characteristics, assess neurological impairment and define stroke severity. The Barthel Index was used to assess the level of dependence in activities of daily living. In addition, the Stroke Impact Scale (SIS) 3.0 Uganda version was used to assess the impact of stroke in everyday life as perceived by the individuals in the chronic sample receiving rehabilitation.

**Results:**

The mean age of the acute/subacute sample was 49 years and 81% had moderate or severe stroke. The mean age of the chronic rehabilitation group was 53 years and 58% had mild stroke. Time since onset in the acute sample was between 2 days and 3 weeks, and time since onset for the chronic sample varied between 3 months and 3 years. Strength, hand function and participation were the most impacted SIS domains in the chronic sample.

**Conclusion:**

People with severe and moderate stroke were more likely to be admitted to Mulago Hospital. The mean age in the study sample was lower than that in high-income countries. Further knowledge is needed regarding the impact of stroke to develop guidelines for stroke rehabilitation interventions feasible in the Ugandan healthcare context in both rural and urban areas.

## Introduction

Stroke is the second leading cause of death and the third highest cause of disability globally (Johnston, Mendis & Mathers 2009). Although more than 85% of people who suffer from stroke are living in low- and middle-income countries, most research, as well as most prevention and intervention initiatives, is facilitated in high-income countries (Feigin et al. 2009; Johnston et al. 2009; Strong, Mathers & Bonita 2007). The incidence of stroke in Africa has increased in the last two decades (Owolabi et al. 2015). This increase has been attributed to lack of knowledge regarding stroke and poor control of the increasing stroke risk factors among the population (Akinyemi et al. 2009; Mensah 2008; Njoku & Aduloju 2004; Owolabi et al. 2015). Moreover, infectious diseases are more often prioritised in low-income countries; consequently, inadequate resources are allocated for stroke prevention and rehabilitation (Johnston et al. 2009; Owolabi et al. 2015).

No studies are available regarding assessing the impact of stroke among people in Uganda (Chin 2012), and this knowledge is essential for the development of care and rehabilitation interventions.

People with fatal stroke or mild stroke often do not present to hospitals (Owolabi et al. 2015). This introduces a bias in the registration of new cases of stroke as well as in reliable reports of death after stroke. Subsequently, studies on population-based groups of people with stroke are lacking in Uganda and other sub-Saharan African countries. A substantial number of patients with stroke receive their initial care in Mulago National Referral Hospital in Kampala; the admission records reveal that every month 22–30 people receive their initial care after stroke onset in the Neurology Ward (Chin 2012; Kwarisima et al. 2014). Because of long distances from their homes, coupled with poor public transport and heavy traffic congestion in Kampala city, many people with stroke experience the onset of neurological symptoms several days before presenting at Mulago Hospital (Chin 2012). As a result, the situation of people living with the consequences of stroke in Uganda is, on the whole, sparsely explored (Chin 2012), while stroke mortality is 44% (Kwarisima et al. 2014). The proportion of people with stroke who attend rehabilitation at Mulago Hospital has been reported to be low and is probably because of challenges of transport from home to the rehabilitation centre, lack of appreciation of the value of rehabilitation and challenges with resources both at family and hospital levels. Thus, it is very important to explore people’s perceived impact of stroke in their everyday life and the need for rehabilitation after the acute phase of stroke, of which there is very little knowledge.

We know from previous studies conducted in high-income countries that the impact of stroke on the individual is always unanticipated and the impact, resulting in activity limitation and poor life satisfaction, can be devastating (Lai et al. 2003). The impact of stroke for the individual can be assessed by the degree of disability, for example ability to perform daily activities such as self-maintenance, household chores and mobility, which often persists for a long time (Norrving & Kissela 2013; Palmer & Glass 2003).

Research has shown that age, gender, dependency in activities in daily living (ADL), lack of social support and medical or psychological factors may affect the impact of stroke (Nichols-Larsen et al. 2005). Old age and co-morbidities such as diabetes mellitus may be associated with low-activity performance in persons with stroke (Lai et al. 2002).

Standardised assessment instruments can be utilised to reliably identify people’s need for rehabilitation after stroke. Tistad et al. (2012) found that the perceived needs of people with stroke did not correlate completely with that assessed by healthcare professionals using established standardised instruments. Therefore, the perceptions of the individuals with stroke should also be explored and addressed by healthcare professionals.

In Uganda there is a lack of knowledge regarding how people with stroke perceive the impact of stroke on their lives (Nakibuuka et al. 2014). This can be assessed using the Stroke Impact Scale (SIS), a recommended measure for use in rehabilitation research that was developed from the perspective of, and with input from, patients with stroke, caregivers and health professionals with stroke expertise (Duncan et al. 2003). The SIS incorporates meaningful domains of functioning and health-related quality of life into one self-reported questionnaire. The SIS instrument has been culturally adapted, translated into the Luganda language and psychometrically tested in the Ugandan culture (Kamwesiga et al. 2016). The results indicated that the SIS was a valid instrument (SIS 3.0-Uganda) that could be used for evaluating the perceived impact of stroke. Therefore, this study focused on the clinical characteristics and perceived impact of stroke in order to generate knowledge about the need for rehabilitation by people with stroke in Uganda.

### Study objectives

The objectives of this study were to explore and describe the clinical characteristics and functioning during the acute/subacute phase and the chronic phase, as well as the impact of stroke on everyday life during the chronic phase in stroke survivors in central Uganda.

### Specific aims

The following were the specific aims of the study:

- to describe the clinical characteristics and functioning of people with acute or subacute stroke admitted to Mulago National Referral Hospital in Kampala
- to describe the clinical characteristics, functioning and perceived impact of stroke in a sample of people receiving rehabilitation in the chronic phase after stroke.

## Methods

A cross-sectional study design was used.

### Context for the study

Mulago Hospital is the largest public hospital and primarily receives severely ill patients, including stroke patients, from all regions of Uganda (population 34.9 million). The hospital is situated 3 km from Kampala city centre (population 1.6 million). For further rehabilitation, persons with stroke can attend as outpatients at the Stroke Rehabilitation Centre just outside Kampala and at the Mulago Hospital Physiotherapy Department located in Mulago Hospital. At the time of the study, these were the only units providing rehabilitation to people with stroke after discharge from hospital.

### Participants

The inclusion criteria for the acute and the rehabilitation samples were as follows: (1) stroke diagnosis in medical records, first or recurrent, confirmed by a computed tomography (CT) scan or clinical assessment, (2) age not more than 75 years, (3) no psychiatric diagnosis, (4) able to understand and respond to instructions in English or Luganda, (5) admitted to the Neurology Ward for treatment for acute stroke or (6) 3 months since onset but less than 3 years post-stroke for the chronic sample receiving rehabilitation. The participants in the acute sample were consecutively identified following admission to the Neurology Ward within 1–3 weeks after stroke onset. Participants in the chronic sample were persons with stroke who were outpatients at the Stroke Rehabilitation Centre or at the Mulago Hospital Physiotherapy Department.

### Data collection and instruments

A protocol was developed to collect demographic data and clinical characteristics including age, gender, marital status, handedness, number of children, work status, level of education and housing as well as type of stroke, side of body affected by stroke and rehabilitation received. Data were collected from September 2013 to April 2015. The first author and a trained research assistant provided both oral and written information about the study to the participants after which they were invited to participate and to sign a consent form. People with stroke who were not able to sign because of current disability could authorise a caregiver to sign on their behalf. Information on stroke diagnosis and type of stroke was collected from the medical records. Face-to-face interviews were conducted to collect the information on demographic data and clinical characteristics from both the acute/subacute and the sample receiving rehabilitation. The face-to-face interviews were conducted where it was appropriate for the participants to meet the data collector: in the hospital, in the rehabilitation unit or in the participant’s home. The interviews lasted for 45–60 min.

Scandinavian Stroke Scale (SSS) was used to collect clinical characteristics, assess neurological impairment and define stroke severity in three categories: mild (43–58), moderate (26–42) and severe (0–25) (Askim et al. 2016; Christensen, Boysen & Truelsen 2005; Luvizutto et al. 2012). The SSS is designed to give a score based on the level of consciousness, eye movement, orientation, speech, hand and leg movement, gait and facial paralysis. The SSS total score and scores on individual items are reported. The SSS has been used extensively in clinical trials and has been shown to have high inter-observer reliability (0.93) and high concurrent validity (0.94–0.98), especially when performed face-to-face (Barber et al. 2003). The SSS scores range from 0 to 58 points, where 0 is severe and 58 is the total score.

Barthel Index (BI) was used to assess the level of dependence in activities of daily living (ADL) (Mahoney & Barthel 1965). Barthel Index assesses 10 items of ADL: feeding, bathing, grooming, dressing, bowel control, toileting, transferring from chair to bed and back, walking on level surface, and ascending and descending stairs. Item scores are summarised to give a range of scores from 0 (totally dependent) to 100 (totally independent). The BI is widely used and has been shown to have high reliability and validity when used for people with stroke (Lai et al. 2002).

The BI has been shown to be so sensitive that it can detect the onset of a need for assistance, which is of high clinical relevance (Wade & Collin 1988). ADL abilities were evaluated by identifying the total score of BI and then classified into three categories: independent (91–100), with help (60–90) and dependent (≤59). Dependent was defined as the need for personal assistance and independent was defined as independent activity performance with or without the use of assistive devices (Gill, Williams & Tinetti 1995).

The Stroke Impact Scale 3.0 (SIS) was designed to assess the perception of the individual with stroke on functioning in everyday life in eight domains: strength, hand function, ADL/instrumental activities of daily living (IADL), mobility, communication, emotion, memory and thinking, and participation. The SIS version 3.0 includes 59 items within these eight domains (Duncan et al. 2003). The SIS aggregated score ranges from 0 to 100; the higher the score, the lower the perceived impact of stroke, that is, fewer problems in everyday life. The SIS 3.0 also includes a question to assess participants’ global perception of recovery presented in a vertical analogue scale ranging from ‘0 = no recovery to 100 = full recovery’. The SIS 3.0 Uganda version (Kamwesiga et al. 2016) was used to assess the perceived impact of stroke in the chronic rehabilitation sample only because some of the items in SIS concern stroke survivors’ perceptions no earlier than 4 weeks after stroke onset.

### Data analysis

Descriptive statistics were used to describe the acute and chronic samples (Allen et al. 2012) regarding demographic and clinical characteristics, functioning and perceived impact of stroke. The SIS aggregated scores in each domain were generated using an algorithm (Duncan et al. 1999). The results in the chronic sample receiving rehabilitation on reported impact of stroke were described based on these aggregated scores.

### Ethical considerations

Ethical approval was granted by the ethical review committee of the Uganda National Council for Science and Technology (ethics approval number: HS 703). Ethical approval for the study was obtained and renewed yearly.

## Results

A total of 120 participants with stroke were included in the study. At the time of the study, 58 participants were receiving acute care on the Neurology Ward and 62 participants were undergoing rehabilitation at the Stroke Rehabilitation Centre or Mulago Hospital Physiotherapy Clinic. It was not possible to contact participants receiving acute care on the Neurology Ward for follow-up after discharge; the attrition rate was almost 100%. The participants’ demographic characteristics are shown in Table 1 and their clinical characteristics are shown in Table 2 for both samples.

**TABLE 1:** Socio-demographics of the study participants.

Participants with stroke	Included at stroke unit†			Included at rehabilitation unit‡		
	*n*	SD	%	*n*	SD	%
**Age (years)**						
Mean	49	14.2	-	53.1	14.2	-
**Gender**						
Men	22	-	38	29	-	47
Women	36	-	62	33	-	53
Born in Uganda	57	-	98	58	-	94
Not born in Uganda	1	-	02	4	-	06
**Number of children**						
Mean	5.6	3.8	-	5.9	3.4	-
No data	2	-	-	4	-	-
**Number of children living at home**						
Mean	3.0	2.6	-	2.9	2.6	-
No data	12	-	-	18	-	-
**Level of education**						
No education	4	-	07	4	-	07
Primary school	27	-	47	17	-	27
Secondary school	19	-	33	22	-	36
Tertiary school	6	-	10	8	-	13
University	2	-	03	9	-	14
No data	-	-	-	2	-	3
**Working before stroke**						
Yes	40	-	67	45	-	73
No	18	-	33	17	-	27
**Housing**						
Own house	33	-	57	46	-	74
Rented	11	-	19	9	-	14
Own apartment	8	-	14	1	-	02
Staff quarters	4	-	07	2	-	03
Living with relative	2	-	03	4	-	7
**Marital status**						
Married, living together	31	-	53	39	-	63
Married, not living together	6	-	10	1	-	02
Single/widowed	21	-	37	22	-	35
**Used assistive aids before stroke**						
Yes	02	-	03	0	-	0
No	56	-	97	62	-	100
**Source of family income**						
Small business	23	-	40	22	-	36
Employed	13	-	22	18	-	29
Temporary employment	16	-	28	15	-	24
No data	06	-	10	07	-	11

† , *n* = 58; age range 16–74;

‡ , *n* = 62; age range 19–75.

SD, standard deviation.

**TABLE 2:** Clinical characteristics of the participants (*n* = 120).

Participants with stroke	Included at stroke unit†		Included at rehabilitation unit‡	
	*n*	%	*n*	%
**Dependency by Barthel Index (BI)**				
Dependent (0–59)	51	88	17	27
Moderately dependent (60–90)	2	3	26	42
Independent (91–100)	5	9	19	31
**Stroke severity as per SSS**				
Mild (SSS=43–58)	7	12	36	58
Moderate (SSS=26–42)	24	41	21	34
Severe (SSS=0–25)	23	40	2	3
No data	4	7	3	5
**Hemisphere**				
Right	25	43	31	50
Left	33	57	31	50
**Type of stroke**				
Haemorrhage	9	15	6	10
Infarct	25	43	33	53
Unspecified stroke	1	2	2	3
No data	23	40	21	34
**SSS: Handedness**				
Right	52	90	58	94
Left	6	10	4	6
**SSS: Speech production**				
No aphasia	19	33	40	64
Limited vocabulary	17	29	13	21
Aphasic	18	21	6	10
No data	4	7	3	5
**SSS: Hand motor power**				
Normal strength	3	5	10	16
Motor impairment	13	24	28	45
Paralysis	37	64	21	34
No data	4	7	3	5
**SSS: Gait**				
Walks 5 m without aid	5	09	22	36
Walks with aid	1	2	16	26
Walks with aid & help of another person	7	12	09	15
Unable to walk	41	71	11	18
No data	4	7	3	5
**SSS: Orientation**				
Oriented (correct for time, place and person)	29	50	51	82
Disoriented	25	44	8	13
No data	4	7	3	5
**SSS: Leg Motor Power**				
Normal strength	2	4	10	16
Motor impairment	17	29	39	63
Paralysis	35	60	10	16
No data	4	7	3	5

† , *n* = 58;

‡ , *n* = 62.

SSS, Scandinavian Stroke Scale.

The majority of the participants in the acute sample were admitted to the Neurology Ward 1–2 days after stroke onset. The acute sample had a mean BI score of 51, and 88% were dependent in the performance of ADL. The majority (81%) of the acute sample had moderate to severe stroke according to the SSS, with 40% having severe post-stroke neurological impairment.

According to BI scores, 31% of the chronic sampe receiving rehabilitation were independent in ADL, while 42% were in need of some help and 27% were dependent in ADL. The majority (58%) of participants in the chronic group had mild neurological impairment according to the SSS and 3% had very severe neurological impairment.

Table 3 presents the perceived impact of stroke (SIS) in the chronic rehabilitation sample. Participants perceived the greatest impact of stroke in the domains of strength, participation and hand function, with domain scores of 44.7, 36.5 and 28.8, respectively. Furthermore, hand function had the lowest median score of 10. The domains of communication and memory and thinking had the highest mean scores of 84.4 and 85.1, respectively. Self-reported recovery ranged from 40 to 90, with a median of 60.

**TABLE 3:** The Stroke Impact Scale 3.0 aggregate domain scores and self-rated recovery (*n* = 62).

SIS domains	Mean domain score	SD	Median domain score	Range domain score
Strength	44.7	27.6	37.5	0.0–100.0
Memory and thinking	85.1	19.4	92.9	21.4–100.0
Emotions	64.3	15.6	63.9	27.8–100.0
Communication	84.4	22.9	96.4	0.0–100.0
ADL/IADL	55.4	27.2	52.5	17.5–100.0
Mobility	59.0	25.9	62.5	5.6–100.0
Hand function	28.8	34.9	10.0	0.0–100.0
Participation	36.5	21.1	34.1	4.6–91.0
Stroke recovery	59.0	14.0	60.0	40.0–90.0

SIS, Stroke Impact Scale; SD, standard deviation; ADL/IADL, activities in daily living/instrumental activities of daily living.

## Discussion

Uganda is one of the low-income countries where stroke is becoming a serious public health concern (Chan 2015). This study is the first to describe the clinical characteristics, functioning and perceived impact of stroke in a chronic sample receiving rehabilitation in Uganda. The mean age in the acute and the chronic rehabilitation samples was low, 49 and 53 years, respectively. The majority of the participants in the acute sample had moderate or severe neurological impairment, indicating that this group was more likely to seek medical attention and be admitted to Mulago Hospital in Kampala, which was also found by Lawson (2004).

Although more than half of the chronic sample had had a mild stroke, it is plausible that people with milder impairment might not seek medical advice, as has been suggested by Owolabi et al. (2015). The SIS domains perceived as most impacted by the participants in the chronic rehabilitation sample were strength, hand function and participation.

The mean age of this study population is 49 years, and it reflects previous studies that have shown that stroke affects a young population in Uganda and other sub-Saharan African countries, compared to a mean age of 75 years for stroke in high-income countries (Cossi et al. 2012; Kwarisima et al. 2014). The young Ugandan population has a life expectancy of 58 years and appears to be compromised by HIV and AIDS and other communicable diseases. Greater attention needs to be focused on prevention strategies to target risk factors so that the incidence of non-communicable disabling conditions can be reduced.

More women than men with stroke were included in both the acute and the rehabilitation samples. This gender difference is in agreement with previous reports, which have suggested that women have a higher probability of seeking treatment at cheaper government hospitals (Lawson 2004). Previous studies (Bwala 1989; Garbusinski et al. 2005; Kwarisima et al. 2014; Njoku & Aduloju 2004) have also shown that women have more severe strokes that require hospital admission. Furthermore, a study from Tanzania showed that the number of women affected by stroke was higher than that of men, and women were generally more disabled and dependent than men (Walker et al. 2000). In contrast, study findings from Nigeria and Tanzania suggest that males with stroke outnumber their female counterparts (Garbusinski et al. 2005; Walker et al. 2000).

In this study, ischaemic stroke was found to be the most common type of stroke, which is in agreement with an earlier study in Uganda (Kwarisima et al. 2014). The same result was reported in a study from Tanzania at Muhimbili National Referral Hospital (Mlay & Bakari 2010) but is in contrast to the findings from studies in Nigeria (Njoku & Aduloju 2004) where haemorrhagic stroke has been reported to be more common than ischaemic stroke.

The result from SIS 3.0 Uganda version showed that strength, hand function and participation were the most impacted SIS domains with low mean scores for the rehabilitation sample. Our findings concur with those from other studies using the SIS (Guidetti et al. 2014; Lai et al. 2002; Nichols-Larsen et al. 2005), but these domain scores were even lower in the Ugandan sample. The mean scores in other SIS domains such as emotion, ADL/IADL and mobility were lower than in a Swedish study (Guidetti et al. 2014), although the findings showed trends similar to previous studies (Carod-Artal et al. 2008; Duncan et al. 2003). A plausible reason for the reported high impact on hand function and participation could be that people with stroke who were referred to physiotherapy in the subacute phase had impaired hand function and decreased physical strength. Hand function is necessary for performing activities in almost all contexts. In the cultural context of this study, hand function might be even more vital than in high-income countries because more households in Uganda earn their living from work for which physical strength and fine motor ability are essential, such as for gardening, construction work and handicrafts; this is also shown in a study from Rwanda (Urimubenshi 2015). Any hand impairment could have been perceived as a serious restriction to participation in daily life. There was a low perceived impact on memory and thinking and emotion domains. One explanation could be that such impairments may be less obvious in everyday life than perceptions of physical limitations (Lai et al. 2002).

## Methodological considerations

One strength of this study, even though the study samples were small, was the similarity of the demographic characteristics with those in previous studies on people with stroke in other sub-Saharan countries (Bwala 1989; Cossi et al. 2012; Garbusinski et al. 2005; Walker et al. 2000). In addition, the mean age and other characteristics are in line with results previously reported for a group of people with stroke from Uganda (Nakibuuka et al. 2014). However, the results should be translated with caution to the broader population of people with stroke in Uganda and sub-Saharan Africa as this study involved a small number of participants in an urban and peri-urban sample. Moreover, the study applies commonly used and well-established assessment instruments such as the BI, SSS and SIS. Furthermore, with regard to stroke severity, the measurements represent the patient perspective, that is, a patient-reported outcome measure (SIS), as well as the views of healthcare personnel (SSS), functioning in personal ADL, and the BI. An additional strength of the study was that all data were collected in face-to-face interviews. Written self-reports would not have been feasible for all participants because of language and literacy complications. Moreover, data collection for the rehabilitation sample was conducted in the participants’ homes, enabling validation of the participants’ responses.

One limitation of the study is the use of two separate samples in a cross-sectional design instead of a prospective longitudinal design which could have provided information on the stroke survivors’ process of return to everyday life after stroke and the need for rehabilitation to regain functioning. Furthermore, another limitation regarding the representativeness of the included samples is the use of the inclusion criteria: ‘age not more than 75 years’ and ‘less than 3 years post-stroke’. These criteria might have caused a bias by excluding persons with stroke having other clinical problems that impacted their functioning even if both samples were relatively young in comparison to samples with stroke in high-income countries.

The original study design involved inclusion of persons with stroke from the Neurology Ward, conducting baseline assessments and 3-month follow-up after stroke onset. However, patients at Mulago Hospital primarily had severe stroke, and it was difficult to trace people with less severe stroke. Very few of the participants included in the acute group were accessible for follow-up. Many were deceased; others returned to their rural homes and could not be contacted. The attrition rate was therefore close to 100%. Because of the high attrition rate, we decided to alter the design to a cross-sectional study involving two separate samples. By including participants who were receiving rehabilitation, we were able to collect information on the perceived impact of stroke from people with less severe stroke who had progressed from the acute phase after stroke.

This study is the first of its kind and should be seen as an initial step in building knowledge regarding the impact of stroke in daily living in Uganda. Thus, this explorative work should be pursued and evaluation of people with stroke and their families using relevant, culturally adapted assessments of their situation should be continued. More knowledge about the impact of stroke is essential for the development of guidelines for stroke care in Uganda. This would include rehabilitation interventions that are feasible in the Ugandan healthcare context in both rural and urban areas. Furthermore, future research should focus on interventions that are relevant, accessible and culturally acceptable to those who need them most, utilising available and accessible resources within the healthcare context, such as the use of mobile phones, to provide basic rehabilitation services after stroke (Kamwesiga, Tham & Guidett 2017).

## Conclusion

People with severe and moderate stroke were more likely to be admitted to the Neurology Ward at Mulago Hospital, Kampala, Uganda. The mean age of the study sample was much lower (mean 49) than the mean age of stroke in high-income countries. The domains perceived as being most impacted by the stroke were strength, hand function and participation in daily life. There is a need for further knowledge about the impact of stroke to develop guidelines for stroke care including rehabilitation interventions feasible in the Ugandan healthcare context in both rural and urban areas.
