# Corrigendum: The impact of stroke on people living in central Uganda: A descriptive study

**DOI:** 10.4102/ajod.v8i0.606

**Published:** 2019-10-10

**Authors:** Julius T. Kamwesiga, Lena K. von Koch, Gunilla M. Eriksson, Susanne G.E. Guidetti

**Affiliations:** 1 Department of Neurobiology Care Sciences and Society, Karolinska Institute, Sweden; 2 Occupational Therapy School, Institute of Allied Health and Management Sciences - Mulago, Uganda; 3 Department of Neurology, Karolinska University Hospital, Sweden; 4 Department of Neuroscience, Rehabilitation Medicine, Uppsala University, Sweden

In the version of this article published earlier, the surname of the second author, Lena K. von Koch, was inadvertently misspelt as ‘von Kock’. The second author’s surname should have appeared as ‘von Koch’ throughout the author list and ‘how to cite’ information section. This correction does not alter the study’s findings of significance or overall interpretation of the study results. The author apologises for any inconvenience caused.

